# *BackProx*: Secure Backscatter-Assisted Proximity Detection for Passive Keyless Entry and Start Systems

**DOI:** 10.3390/s23042330

**Published:** 2023-02-20

**Authors:** Hoorin Park, Jeongkyu Hong

**Affiliations:** 1 Department of Information Security, Seoul Women’s University, Seoul 01797, Republic of Korea; 2 Department of Computer Engineering, Yeungnam University, Gyeongsan 38541, Republic of Korea

**Keywords:** backscatter, frequency hopping, secure proximity detection, passive keyless entry and start system, relay attack

## Abstract

A passive keyless entry and start (PKES) system is an electronic lock for an automobile that provides the great convenience of opening the door when the user is in proximity. However, the system suffers from relay attacks. Recent studies revealed that relayed signals result in valid packets that are sufficient to unlock doors. In particular, the adversary causes proximity errors by injecting a certain time delay before relaying to manipulate the phase rotation in the response signal. To this end, we present a novel relay-resilient proximity detection solution, *BackProx*, which uses pseudo-random frequency hopping with the assistance of a reference backscattering device. Since the relay adversary transmits the relayed signals from the key fob at long distances, the signals should propagate over longer distances, resulting in inevitable significant phase rotation with different frequencies. Inspired by this finding, *BackProx* uses an additional backscattering device to ensure the proximity of the key fob using the invariant characteristics of radio frequency signals in the physical layer (i.e., phase rotation). Our evaluation demonstrates the effectiveness of *BackProx* in resisting three types of relay attacks. The results show that it achieved a 98% true positive rate at close range and a 0.3% false positive rate at long range.

## 1. Introduction

Proximity detection is widely used in a variety of applications, such as toilet flush sensors, parking assistance, payment systems, transportation ticketing, physical access control [1], contagion tracking [2], and PKES systems [3]. In particular, in PKES systems, car doors are unlocked automatically when the vehicle detects the key fob in the vicinity. PKES systems allow users to open their cars without active user interaction with the keys, and the only thing they need to do is get close to their cars. Unlike other applications, the PKES system requires a more accurate and secure proximity detection technique since it is related to property directly. With the prevalence of keyless systems, the significance of secure proximity detection is increasing.

Today, modern cars that use PKES systems rely on challenge–response approaches [3,4]. Since PKES systems do not require user interaction, the user cannot notice whether their keys are working or not. Hence, the attacker can freely access the key and relay the signal responses to the car to purport that the key is in the vicinity. This is an example of a relay attack. Although the attacker lacks knowledge on how to respond to the challenge, he or she can argue the proximity and pass authentication by only relaying the signals between the car and the key.

Unfortunately, most proximity detection techniques are inherently insecure, because no matter how well designed the cryptographic protocols in RFID devices are, they cannot avoid the threat of distance-decreasing attacks [5,6]. Since these attacks do not require the messages to be modified or decrypted, the prover and verifier may not be aware of such attacks [7,8]. Even worse, the widespread adoption of the Internet of Things has led to a proliferation of proximity detection applications without proper consideration of the threat posed by wireless distance-decreasing attacks, such as relay attacks [9,10]. Therefore, it is obvious that the design of a secure proximity detection system is necessary [11].

Conventional proximity detection techniques can be classified as follows. First, several techniques use distance-bounding protocols using the measured received signal strength indication (RSSI) or time of flight (ToF). These mainly depend on changes in the physical layer’s radiometric characteristics due to signal propagation. However, an attacker can deceive the communication systems with an amplified signal strength or relayed signals [3,12]. To detect the relayed signals from a distance, a response timeout (i.e., RTT) is given by distance-bounding protocols [13,14,15,16,17]. However, the precision of the time estimate depends on the system’s bandwidth. For example, commercial proximity detection transceivers typically use sampling rates of up to 2 Mbps. This restriction leads to a large spacing of 150 m, given the speed of light. This space is considered a sufficient distance for an attacker to commit relay attacks. Furthermore, D. Celiano pointed out that overclocked electromagnetic carriers can speed up the computation of an NFC card and allow relay attacks within the timeout period [18].

Typically, short-range communication technologies such as radio frequency identification (RFID) and near-field communication (NFC) can be used as inherent proximity detection techniques. Based on their limited communication ranges, they are common communication technologies for proximity detection systems. Unfortunately, relay attacks on these technologies easily compromise the systems, even though they use cryptographic approaches [12,19,20,21].

Another method is a context-based co-presence detection technique based on the fact that the vehicle and the key fob should share the same context information, such as the GPS location, humidity, temperature, barometer, and altitude [22,23]. Since the technique requires the exchanges of information obtained from additional sensor modalities, both cars and key fobs have to pay an extra cost for the additional hardware, power, and time to collect the environmental factors for each detection attempt. This restriction discourages market entry.

In this context, we propose *BackProx*, a new secure proximity detection technique that uses an additional backscatter tag to resist relay attacks. *BackProx* is basically based on multi-carrier phase information. In our attack models, the attacker manipulates the phase information in a signal and relays it to the verifier without any knowledge of the cryptographic primitives implemented. Existing carrier phase-based proximity detection techniques are vulnerable to phase-manipulating attacks [24]. Briefly, the distance estimate from the phase information is vulnerable to phase manipulation attacks because the estimated distance can be decreased or increased by the attacker.

**Figure 1:**
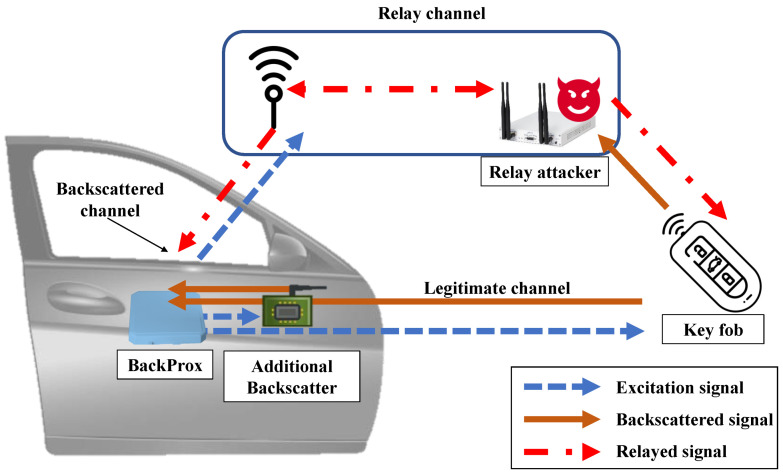
BackProx system model and relay attack. *BackProx* aims to defend against relay attacks, in which an attacker obtains a legitimate signal from a key and forwards it to a verifier to pass the proximity detection system.

To address this vulnerability, we designed a novel relay-resilient proximity detection scheme called *BackProx*. Instead of using a single key fob, *BackProx* uses an additional backscatter tag to prove proximity, as shown in Figure 1. The additional tag provides evidence of proximity in relay attacks. We do not estimate the distance between the verifier and prover but compare the distances among the additional tag, verifier, and prover. To purport proximity, the attacker would change the phase information from the response signals. The change causes estimation errors from the tag to the verifier and the prover to the verifier. *BackProx* then calculates the difference between the two phase values measured by the verifier, which effectively mitigates the effects of phase manipulation. Our evaluation demonstrates that *BackProx* can resist the relay attacks effectively and achieve a 98% true positive rate at close range and a low false positive rate of 0.3% at far range.

The main contributions of this paper are as follows:

- We present *BackProx*, a novel and secure proximity detection system which uses an additional backscatter tag to provide evidence of proximity.
- We investigate concrete attack models for relay attacks and examine how *BackProx* defends against these attacks effectively. Our security analysis is theoretical and aims to provide the mechanisms behind the resistance of *BackProx* to these attacks.
- We provide analytic evaluation of *BackProx* and demonstrate its proximity detection performance.

The rest of this paper is organized as follows. In Section 2, we review the previous work on proximity detection. Section 3 presents a system model for *BackProx* and includes the conventional phase-based proximity detection schemes in the literature. The concrete attack models against phase-based proximity detection schemes are explained in Section 4. Section 5 describes the technique details of *BackProx*. We present an analytical evaluation of *BackProx* that provides theoretical analysis of how *BackProx* resits relay attacks and its proximity detection performance in Section 6. Section 7 addresses the limitations of *BackProx* and suggests potential countermeasures to improve security, as well as a future work direction. Finally, we conclude this paper in Section 8.

## 2. Related Work

Recently, there has been interest in using the characteristics of radio frequencies (RFs) for proximity detection. In [25], the authors utilized their observations on fluctuations of ZigBee signals in the near field. However, the approach does not address the threat of relay attacks. HODOR [26] is based on an RF fingerprinting approach that verifies an RF’s features using a trained classifier. While the method is effective, it requires the extraction of an RF’s features, which are highly specific to the current environment, devices, and other factors and which can be time-consuming or may not be feasible for everyday users. Additionally, HODOR assumes that the key fob is an active transmitter with an oscillator. Since a passive RFID-based key fob backscatters the ambient RF signals from a dedicated carrier emitter instead of emitting the carrier frequency itself, the features introduced in HODOR cannot be applied directly. In this context, we propose a new relay-resilient proximity detection method, *BackProx*, which uses the RF’s characteristics but does not require any training sets.

Other approaches have incorporated machine learning techniques to mitigate relay attacks [27,28]. The proposed methods utilize security features including key fob acceleration, signal strength, location, and time to achieve a high accuracy rate of 99.8%. However, these approaches rely heavily on a time-consuming and labor-intensive feature data collection phase to establish a dataset for a legitimate user. For example, in [27], the authors collected 3 months of key fob logs and 300 records of a driver, with each record taking 5 min to gather. Additionally, not all ambient condition sensors are available in RFID-based systems.

In SNAP [29], the authors proposed a proximity detection method that uses a single antenna. The method is based on the observation that mismatching of WiFi signals will occur at the same part in the preamble, which should appear repeatedly in each packet. When the target device is close, the received parts do not match each other due to near-field effects. However, this mismatching can also be caused by an adversary. The authors also discussed the attack and propose a countermeasure that uses a trusted device located far away to verify the proximity. If the target is legitimate, then the far away device should observe the matching in the preamble, which can help confirm the proximity. While SNAP presents a practical method for proximity detection, the requirement for an additional trusted device may not be feasible for all users.

Other recent studies, such as those on Move2Auth [30] and RF-Rhythm [31], proposed approaches for device authentication that are based on a change in RF characteristics. These methods use changing patterns in RF characteristics to authenticate users who are in the vicinity. However, both approaches require user interaction, which limits their usability and practicality for proximity detection applications such as PKES. To provide practical proximity detection applications, it is important to design systems that do not require user interaction and operate in a seamless manner.

BackProx uses a multi-tag based technique to provide a practical solution for secure proximity detection in existing systems. In this context, there are several studies that proposed security enhancement methods based on multi-tag technology, such as Butterfly [32], SCBF [33], Tagora [34], and Hu-Fu [35]. These approaches aim to minimize environmental sensitivity [32] or use collision signals [33,34] or inductive coupling [35] as a kind of fingerprint to authenticate backscattering devices. While these methods are effective at providing resistance to traceability, impersonation attacks, and environmental sensitivity, they require the users to have redundant tags or reduce the authentication range dramatically, which limits the design space for backscattering applications such as PKES systems [3]. *BackProx* has a similar approach to providing security enhancement, but it does not require the user to have additional devices or a limited range, which makes it more practical and convenient for everyday users.

## 3. System Model

Suppose we have a PKES system with a passive RFID-based key fob. When a legitimate user tries to unlock the door by pulling the door handle, the car emits an excitation signal to wake up the key fob. The key fob backscatters the excitation signal and transmits its response message. From this point on, we call the car the verifier and the key fob the prover.

From the perspective of a backscattering communication system, the verifier acts as a reader with prudent power and computation resources. When the verifier transmits a constant wave (CW) as the excitation signal, the tag at the prover backscatters the CW to the verifier with its modulated message. The response message is received at the verifier, and the phase is measured. Then, the phase is expressed as follows [36]:

(1) $$\theta =\left(\theta_{prop}+\theta_{orient}+\theta_{reader}+\theta_{tag}\right)\;\mathrm{mod}\;2\pi .$$

**Figure 2:**
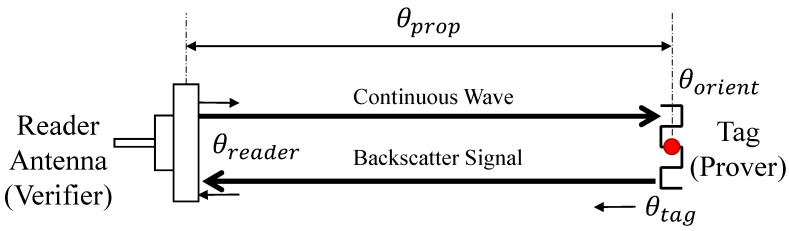
Phase model of backscattering systems. A continuous wave is transmitted from the reader to the tag, and a response backscattering signal is transmitted back to the reader with phase rotation.

At the verifier, the received signal is propagated from the reader’s transmitter antenna to the tag and back to the reader’s receiver antenna as shown in Figure 2.

The phase change caused by the signal’s propagation (i.e., $\theta_{prop}$) can be calculated as $\theta_{prop}=\frac{4\pi }{\lambda }d$, where *d* is the distance between the verifier and prover and λ is the wavelength of the CW. The orientation of the tag also affects the phase information $\theta_{orient}$. It is constant and does not change depending on the frequency. The phase values caused by the impedance of the reader and tag are denoted by $\theta_{reader}$ and $\theta_{tag}$, respectively. Although the previous work shows the impedance changes according to the attached material [37], we assume that the tagged material does not change during the proximity detection process. Thus, the phase change caused by impedance is stable. Now, we can express Equation (1) as follows:

(2) $$\theta =\left(\frac{4\pi }{\lambda }d+\theta_{orient}+\theta_{reader}+\theta_{tag}\right)\;\mathrm{mod}\;2\pi .$$

For simplicity, commercial off-the-shelf (COTS) RFID systems [38] and the existing work [39] often summarize the stable values as a constant *k*. The wavelength λ at constant speed of light *c* is given by $\lambda =\frac{c}{f}$. Therefore, the relationship between the phase and frequency is given by

(3) $$\theta \left(f\right)=\left(\frac{4\pi d}{c}f+k\right)\;\mathrm{mod}\;2\pi .$$

Equation (3) indicates that the measured phase θ(f) changes linearly with the frequency *f*, and the slope of the line is determined by a certain distance *d*.

For multi-carrier phase-based proximity detection, we can use different frequencies such as $f_{1}$ and $f_{2}$. From the difference between $\theta (f_{1})$ and $\theta (f_{2})$, the distance estimate is expressed as

(4) $$d=\frac{c}{4\pi }\cdot \frac{\theta \left(f_{1}\right)-\theta \left(f_{2}\right)}{f_{1}-f_{2}}.$$

Then, if the system estimates the distance *d* to be less than a certain threshold, then it decides the proximity of the prover. However, when an attacker manipulates the measured phase information as he or she wants, this decision can be compromised. The details of the attacks are described in Section 4. In the attacks, *BackProx* uses an additional backscatter tag to provide evidence for proximity. The additional tag also acts as an ordinary backscattering tag in the prover that satisfies Equations (1)–(4).

## 4. Adversarial Model

With the prevalence of low-cost software-defined radio systems, an attacker can easily eavesdrop on, manipulate, replay, and relay radio signals. This means that the attacker has full control over the wireless radio channels used to authenticate the proximity. We posit that the attacker does not know encryption techniques at all. Therefore, this paper focuses on the capability of an attacker that manipulates the radio signals in the physical layer.

The adversarial model is based on the Chess Grandmaster problem [40]. In this problem, a little girl who does not know how to play chess plays two chess games against grandmasters at the same time: one with black and the other with white. Then, by only relaying the moves of each grandmaster, she can defeat at least one grandmaster. If we apply this problem to our model, then an attacker can deceive a verifier by relaying the signals from a prover without any knowledge of implemented cryptographic primitives.

The goal of an attacker is to deceive the proximity detection at a verifier, where the attacker is located close to the verifier and the prover is outside the neighborhood. In particular, our adversarial model focuses on relay attacks that relay the legitimate signals from the prover to pretend to be close to the verifier. Some early papers considered a weaker relay attack model that relays only the messages between a verifier and a prover without any manipulation. However, this weak relay attack model could be defended by basic phase- or ToF-based distance-bounding protocols.

For a stronger adversarial model, we consider three concrete attack models that try to decrease the distance, such as a phase-slope rollover attack, an RF cycle slip attack, and an on-the-fly phase manipulation attack [24]. In the following, we describe the details of these attack models.

### 4.1. Phase-Slope Rollover Attack

For distance estimation based on multi-carrier phase information, a phase difference caused by radio propagation is used. However, if an attacker manipulates the phase information as he or she wants, then this can lead to an estimation error.

In the phase-slope rollover attack, the attacker simply injects the intended phase rotation $\phi_{a}$ to all response signals from the prover. Recall from Equation (4) that the estimated distance *d* is proportional to the phase difference, denoted by Δθ. Since the phase value ranges from 0 to 2π, the maximum value of Δθ is 2π. If the phase value exceeds 2π, it returns to zero (rollover). The attacker can manipulate the phase difference with a time delay Δt before relaying the response signals. A specific time delay is chosen to trigger the phase rollover.

**Figure 3:**
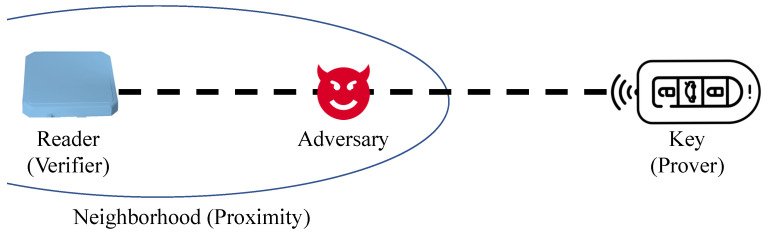
A relay attack model. An attacker is near the verifier, and the prover is located outside the neighborhood of the verifier (called Mafia Fraud).

We assume that the attacker is located close to the verifier and the prover is far away from the verifier, as shown in Figure 3. The goal of the attacker is to reduce the estimated distance from the verifier.

The phase information for the phase-slope rollover attack is derived with the time delay Δt as follows:

(5) $$\theta \left(f\right)=\left\{2\pi (t+\Delta t)f+k\right\}\;\mathrm{mod}\;2\pi ,$$

The round-trip time *t* is the total time it takes for a signal to travel from a transmitter to a receiver and back over a certain distance *d*. Since the verifier uses multi-carrier frequencies, two phase values are used, such as $\theta (f_{1})$ and $\theta (f_{2})$. We express the phase difference as Δθ=2π(t+Δt)Δf, where $\Delta \theta =\theta \left(f_{1}\right)-\theta \left(f_{2}\right)$. The frequency difference between the two phase measurements is denoted by Δf. To estimate the distance, Equation (4) is given by

(6) $$d=\frac{c}{2}\left(t+\Delta t\right)\;\mathrm{mod}\;\frac{c}{2\Delta f}.$$

The modulo operation is added due to the maximum phase difference 2π. If the attacker injects a certain Δt which causes rollover of the modulo operation, then it leads to a reduced distance at the verifier. In general cases, Δf is specified as 2 MHz and presents 75 m as a maximum distance.

**Figure 4:**
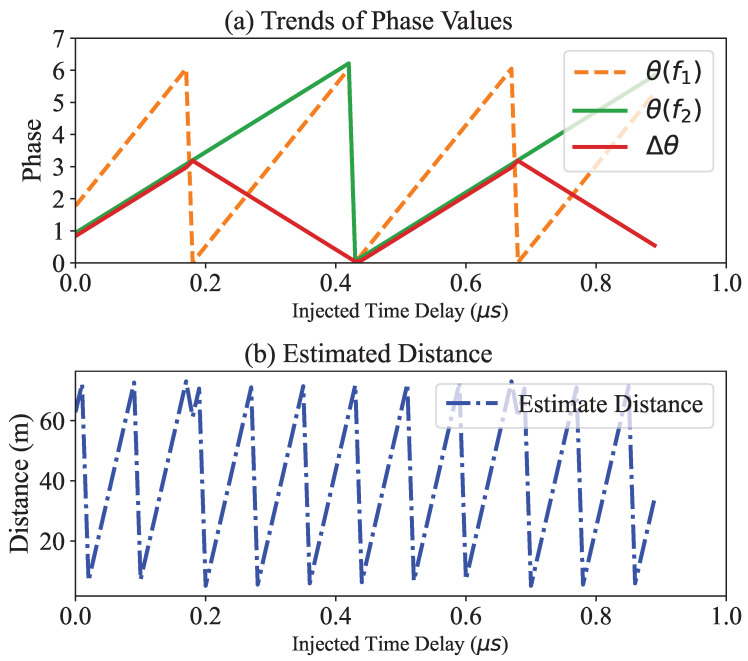
Phase value at the verifier vs. time delay by an attacker. (**a**) The trends of the phase values with two different frequencies. (**b**) The estimated distance calculated from the phase difference, in which the injected time delay causes the rollover effect.

As shown in Figure 4, the graph illustrates the trend of each phase value and the difference depending on the time delay Δt introduced by the attacker, where the distance is 10 m and the frequency hopping size is 2 MHz. This shows that the estimated distance also goes through the rollover effect, depending on the time delay introduced. The rollover effect occurs when the distance estimation exceeds a certain limit and returns to the starting point, which could result in incorrect distance readings and could be used by an attacker to inject a false distance.

### 4.2. RF Cycle Slip Attack

An RF cycle slip attack is similar to the phase-slope rollover attack, except for the values for the time delay. In the attack previously described, the attacker relays all the response signals with the same time delay. On the other hand, in this attack, the attacker manipulates the phase values of individual carrier frequencies. For each frequency $f_{i}$, the attacker selects a different time delay $\Delta t_{i}$.

The phase difference is specified by the time delays $\Delta t_{1}$ and $\Delta t_{2}$ for the frequencies $f_{1}$ and $f_{2}$, respectively. Then, the estimated distance from Equation (4) is given by

(7) $$d=c\left(t+\frac{\Delta t_{1}f_{1}-\Delta t_{2}f_{2}}{f_{1}-f_{2}}\right)\;\mathrm{mod}\;\frac{c}{2\Delta f}.$$

Briefly, the attacker can manipulate the phase information at the verifier using different time delays for each frequency. However, this attack requires more high-cost hardware than the phase-slope rollover attack, since it needs a very high sampling rate to know the individual frequencies before injecting the time delays.

### 4.3. On-the-Fly Phase Manipulation Attack

In on-the-fly (OTF) phase manipulation attacks, the attacker does not simply relay the response signals. Instead, an intermediate frequency (IF) signal is used to manipulate the phase rotation. At the attacker device, the response signal is intercepted and mixed with an IF signal generated by the attacker.

We assume that the response signal from the prover is $s_{p}\left(t\right)$:

(8) $$s_{p}\left(t\right)=A\left(t\right)\cos\left(2\pi ft+k\right)$$

where A(t) is the amplitude modulated by the prover. The attacker receives the response signal and generates an intermediate frequency signal $s_{if}\left(t\right)$:

(9) $$s_{if}\left(t\right)=\cos\left(4\pi ft+\theta_{att}\right),$$

where $\theta_{att}$ denotes the injected phase offset by the attacker.

The attacker uses a mixer to manipulate the phase information in the response signal. After mixing of the two signals in Equations (8) and (9), the manipulated signal is given by

(10) $$\begin{array}{ccc} s_{m}\left(t\right)=s_{p}\left(t\right)\cdot s_{if}\left(t\right) & = & A\left(t\right)\cos\left(2\pi ft+k\right)\cdot \cos\left(4\pi ft+\theta_{att}\right)\\ & = & \frac{1}{2}A\left(t\right)\left\{\cos\left(2\pi ft+\theta_{att}-k\right)+\cos\left(6\pi ft+\theta_{att}+k\right)\right\}. \end{array}$$

The attacker then transmits the manipulated signal through a low-pass filter that removes the high-frequency component. In short, the phase information of the received signal at the verifier can be manipulated with the injected phase offset $\theta_{att}$.

## 5. Methodology

Proving secure proximity detection in wireless systems is not trivial. To be a secure proximity detection technique, we should consider a number of design requirements. We first show the design requirements for secure proximity detection. Then, an overview of the proposed method, *BackProx*, is presented. Next, we elaborate on the design of *BackProx*, which uses an additional backscattering tag with multi-carrier frequencies.

### 5.1. Design Requirements

We provide the design requirements for *BackProx*, which are as follows:

**Requirement 1. The proposed solution should be easily adaptable to existing systems.** The target of *BackProx* is assumed to be currently used proximity-based applications such as PKES systems. On the other side, the solutions based on RF radiometric characteristics cannot be applied to the systems currently in use. The main reason for this is that the solutions require reader hardware such as a software-defined radio that is able to measure an RF’s radiometric characteristics.

**Requirement 2. The proposed solution does not require any changes in the upper layer protocols.** Since the upper layer is not involved in the physical layer, an adversary can easily perform a distance-decreasing attack by relaying a signal, regardless of the encryption technique being used. *BackProx* is designed to provide secure proximity detection while still being able to utilize already-existing protocols. This means that *BackProx* is designed to work seamlessly with the encryption technologies that are currently in place without the need for modifications to be made to the upper layer protocols.

**Requirement 3. The proposed solution should be able to defend against relay attacks with legitimate tag signals.** To ensure secure proximity detection, the solution should be able to defend against the different types of relay attacks described in Section 4. This means that *BackProx* should be designed with the ability to prevent relay attacks and be resilient enough to maintain its performance even when facing these types of attacks.

### 5.2. Overview

The core of *BackProx* is the use of a simple-to-use backscattering tag. *BackProx* consists of three entities: the additional tag and reader, which are owned by the verifier, and the legitimate tag, which is owned by the prover. The additional tag performs the same function as the legitimate tag of the prover, but it is installed by the verifier, which makes it a trusted object at close range. We assume that the additional tag is trustworthy and not compromised by an attacker. The legitimate tag and the additional tag respond in sequence to the signal sent by the verifier, following the existing COTS RFID communication protocol without requiring any additional features to be extracted from the RF signals. This makes it easy to integrate *BackProx* into existing systems without the need to change the upper layer’s protocols.

### 5.3. BackProx Details

Currently, communication protocols for proximity detection in COTS PKES systems involve a wake-up signal from the verifier and a response from the prover, as described in [3]. In addition, to start the engine, proximity detection is carried out through backscattering communication between the vehicle and the RFID tag inside the key fob.

**Figure 5:**
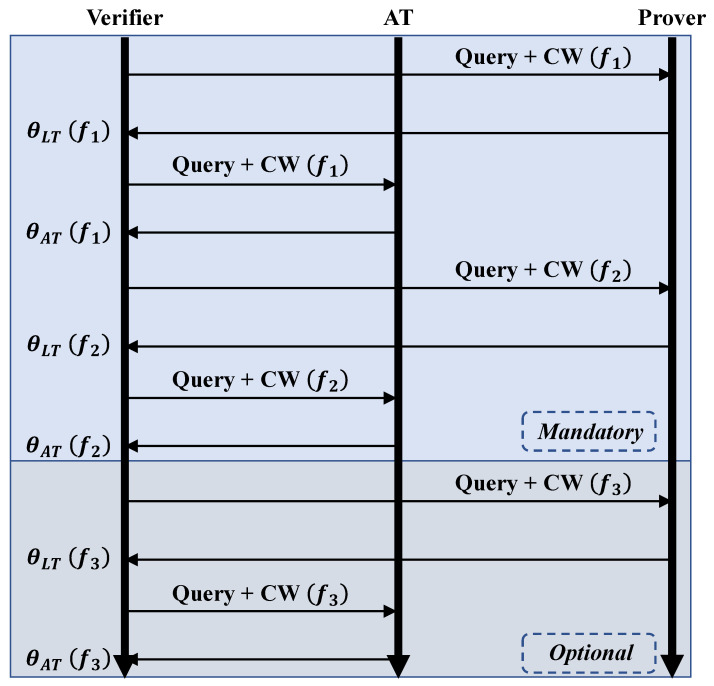
Phase measurement protocol. *BackProx* does not require any modification of the standard protocol or extraction of RF features. This makes *BackProx* a practical and convenient solution for secure proximity detection that can be easily integrated into existing systems.

BackProx maintains communication between the reader and the legitimate tag and also performs supplementary communication with the additional tag. This approach assumes a multi-tag scenario in backscattering networks, and it can easily be implemented in existing systems. Figure 5 illustrates the protocol for sequential tag interrogation.

Essentially, *BackProx* utilizes a multi-carrier phase-based proximity detection method, where each tag (the one inside the prover and the additional tag) is interrogated with multiple frequencies. At this time, the response order of each tag is not specified, and the tag is read according to the reader’s selected command [41]. As shown in Figure 5, multi-carrier phase measurements should be performed through two or more frequencies. Since multi-carrier frequencies are also recommended in FCC 15.247 as a frequency hopping spread spectrum (FHSS) scheme [42], it is applicable in COTS backscatter systems. Additionally, the measurement process can be repeated more than two times using options.

The decision basis for proximity detection in *BackProx* relies on the locations of both the additional tag and the legitimate tag in close proximity. Since the additional tag belongs to the verifier and is fixed in a specific location, the proximity of the additional tag to the legitimate tag indicates that the legitimate tag is within the proximity of the verifier. The reader measures the phase information from each tag for each frequency. The measured phase information is referred to as $\theta_{LT}\left(f\right)$ and $\theta_{AT}\left(f\right)$. The equation for the phase information is given by Equation (3). In this case, LT and AT refer to the legitimate tag and the additional tag, respectively.

To prove the distance similarity of the two tags, *BackProx* uses the phase difference between $\theta_{LT}\left(f\right)$ and $\theta_{AT}\left(f\right)$. Since the response signals from the tags are based on the same frequency *f*, the phase difference is expressed as follows:

(11) $$\Delta \theta \left(f\right)=\theta_{LT}\left(f\right)-\theta_{AT}\left(f\right)=\left\{\frac{4\pi (d_{LT}-d_{AT})}{c}f+k_{N}\right\},$$

where $k_{N}$ denotes the difference between the stable rotations (i.e., $k_{LT}-k_{AT}$). Equation (11) indicates that the phase difference Δθ(f) is proportional to the frequency *f*, and the slope is determined by the distance difference $d_{LT}-d_{AT}$. If the legitimate tag is near the additional tag (i.e., alongside the verifier), then this leads to a very low slope value. Otherwise, the slope will be large. Therefore, the slope can be the basis for determining the proximity.

We denote the slope of the phase function as ∇Δθ(f). *BackProx* obtains the phase information by using two or more multi-carrier frequencies to estimate the slope value. To determine the proximity, we set a threshold $\theta_{th}$. If the slope of the phase difference is above the threshold, then *BackProx* determines that the key fob is in the neighborhood. Otherwise, it determines that the response is not valid. The decision rule can be expressed as

(12) $$\left|\nabla \Delta \theta \left(f\right)\right|\begin{array}{c} {}_{Accept}\\ ≶\\ {}^{Reject} \end{array}\theta_{th}.$$

## 6. Evaluation

In this section, we explain how *BackProx* resists the relay attacks described in Section 4. We also evaluate the performance of *BackProx* with our simulation.

### 6.1. Security Analysis

Here, to validate the security of *BackProx*, we analyze the behavior of *BackProx* under relay attacks.

#### 6.1.1. Phase-Slope Rollover Attack

An adversary simply relays all the signals with a certain time delay, except for the excitation signal from the verifier. The time delay causes phase rollover, where the phase rotation over the limit 2π induces measurement errors at the verifier.

In *BackProx*, the signal from an additional tag is analogous to the response signal from the prover. Recall that the phase information of the tag under a phase-slope rollover attack is expressed as shown in Equation (5). Then, the phase information from each tag is given by

(13) $$\theta_{i}\left(f\right)=\left\{4\pi \left(t_{i}+\Delta t\right)f+k_{i}\right\}\;\mathrm{mod}\;2\pi ,\;\;\;i\in \left\{LT,AT\right\}.$$

Note that the time delay Δt is injected by an attacker to all response signals except for the excitation signal from the verifier (i.e., CW).

Then, we measure the two phase values and calculate the phase difference Δθ(f) between two tags:

(14) $$\Delta \theta \left(f\right)=[4\pi \left\{\left(t_{LT}+\Delta t\right)-\left(t_{AT}+\Delta t\right)\right\}f+k_{N}]\;\mathrm{mod}\;2\pi ,$$

where $k_{N}$ denotes the phase rotation difference between each tag $k_{LT}$ and $k_{AT}$.

Equation (14) shows that the injected time delay is canceled in the phase difference. Since $t_{i}=d_{i}/c$ for i∈{LT,AT}, the equation is re-expressed as Equation (11). Then, if the distance difference is close to zero $\left(d_{LT}\approx d_{AT}\right)$, the slope of the phase function will be very low. Otherwise, under the relay attacks $\left(d_{LT}>>d_{AT}\right)$, the slope will be larger at a predefined threshold. Hence, no matter how much time delay the attacker injects, *BackProx* defends against the phase-slope rollover attack.

#### 6.1.2. RF Cycle Slip Attack

In this attack, the response signal is relayed with different time delays for individual carrier frequencies. In short, instead of Δt, $\Delta t_{f}$ is used to insert the delay for each frequency *f*. Since the legitimate tag and the additional tag respond with the same frequency, the attacker injects the same time delay $\Delta t_{f}$ for the frequency *f*. The phase information is expressed as follows:

(15) $$\theta_{i}\left(f\right)=\left\{4\pi \left(t_{i}+\Delta t_{f}\right)f+k_{i}\right\}\;\mathrm{mod}\;2\pi ,\;\;\;i\in \left\{LT,AT\right\}.$$

Next, when we find the phase difference across the two tags, it also eliminates the injected time delay $t_{f}$ for each frequency *f*. As a result, regardless of the injected time delay, *BackProx* always obtains a refined phase difference function. Then, using the slope of the given function (i.e., ∇Δθ(f)), *BackProx* determines the proximity.

#### 6.1.3. On-the-Fly Phase Manipulation Attack

To manipulate the phase information at the verifier, the attacker intercepts the response signals and mixes them with a specially crafted IF signal. After mixing and transmitting through a low-pass filter, the phase information from each tag is given by

(16) $$\theta_{i}\left(f\right)=\left(2\pi ft_{i}+\theta_{att}-k_{i}\right)\;\mathrm{mod}\;2\pi ,\;\;\;i\in \left\{LT,AT\right\},$$

where $\theta_{att}$ is the injected phase offset from the attacker.

From the two tags, *BackProx* also calculates the phase difference. The difference is expressed as

(17) $$\Delta \theta \left(f\right)=[2\pi \left\{t_{LT}-t_{AT}\right\}f+\theta_{att}-\theta_{att}+k_{N}]\;\mathrm{mod}\;2\pi .$$

Equation (17) indicates that the gradient gets smoother as the prover gets closer to the verifier, and the injected phase rotation is also eliminated. Therefore, it shows that *BackProx* effectively defends against OTF phase manipulation attacks as well.

### 6.2. Simulation

To validate the effectiveness of *BackProx*, we evaluated the performance with simulations in terms of the SNR variable. We assumed that the received signals at the verifier were under additive white Gaussian noise (AWGN) channels. We used $\mathrm{CN}(\mu ,\sigma^{2})$ to denote the circular system complex Gaussian distribution with a mean μ and variance $\sigma^{2}$. This means that the magnitude of the noise followed the Rayleigh distribution, and the phase was uniformly distributed [43].

#### 6.2.1. Simulation Set-up

First, we used a specific scenario where a PKES system determineed the proximity if the prover was closer than 30 cm. The additional tag was located 30 cm from the verifier, and the prover was 30 (close), 60 (close), 200 (near), and 300 (far) cm away. In an ideal case (without noise), we could set the threshold in Equation (12) to $\theta_{th}=\left\{4\pi \left(d_{LT}-d_{AT}\right)\right\}/c=1.26\times 10^{-8}$, where the distance difference was $d_{LT}-d_{AT}=0.3$ and the speed of light was assumed to be $3\times 10^{8}$ m/s.

#### 6.2.2. Impact of the SNR

**Figure 6:**
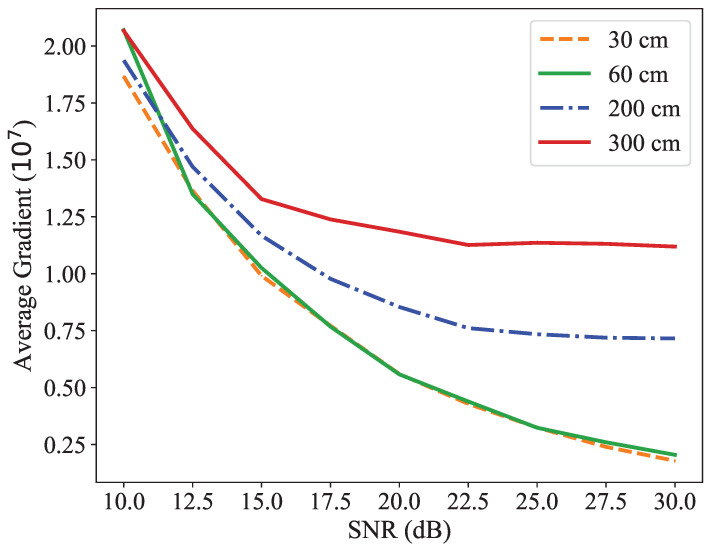
Average gradient of phase difference at difference distances. The gradient can be used as a criterion for proximity detection. When the distance between LT and AT is small, the gradient is very low. When LT is farther away, the gradient becomes very steep. By setting a proper threshold, it is possible to distinguish between these two scenarios and determine the proximity of LT.

To evaluate the effectiveness of *BackProx* for a given scenario, we measured the gradient of phase difference, which is a crucial decision value. Figure 6 illustrates the average gradient of phase difference obtained by varying the signal-to-noise ratio (SNR) from 10 dB to 30 dB. The results show that the average value decreased if the channel was clear. Furthermore, the closer the distance, the lower the resulting value. In proximity detection applications, the SNR is expected to be high when the prover is near the verifier. To achieve a low result value at a high SNR, the distance must be sufficiently small. On the other hand, if the key is far away, even if the channel is clear, a low result value cannot be obtained. Therefore, using the slope of the phase difference between the two tags in *BackProx* can be a reliable way to confirm that the key is in proximity.

#### 6.2.3. Phase Gradient Threshold

**Figure 7:**
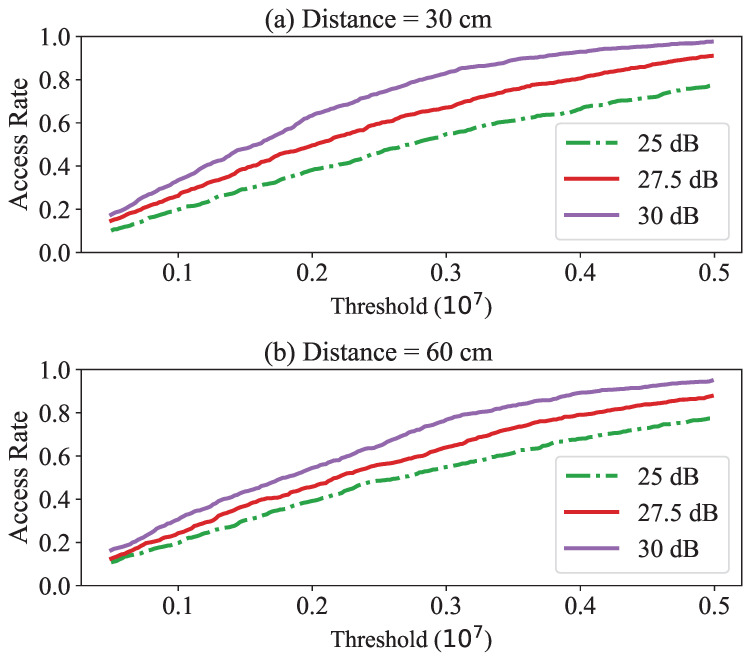
Access rate for different phase gradient thresholds when the distance is close: (**a**) 30 cm and (**b**) 60 cm.

Note that the theoretical threshold for proximity detection at a distance of 30 cm was calculated to be $1.26\times 10^{-8}$. However, as shown in Figure 6, even when the channel was in a clear state, this value was affected by the presence of noise. To determine the appropriate threshold value, we obtained the probability of *BackProx* detecting the proximity at various distances and SNR conditions through simulations. Each rate was the result of performing 1000 trials. Figure 7 shows the rate at which *BackProx* recognized the presence of a prover at distances of 30 cm and 60 cm. In this case, the higher the rate, the better *BackProx* functioned.

At close range, the SNR was high, and there was little interference from noise. Hence, we measured the rates at high SNRs. When the SNR was 30 dB, it was perceived at rates of 98% and 95%. The remaining rates of 2% and 5% were affected by the phase noise, resulting in false rejection. To reduce the false rejection rate, we can increase the threshold. This makes it possible to adjust the range of proximity by adjusting the threshold, because the accuracy improves as the key approaches the verifier.

**Figure 8:**
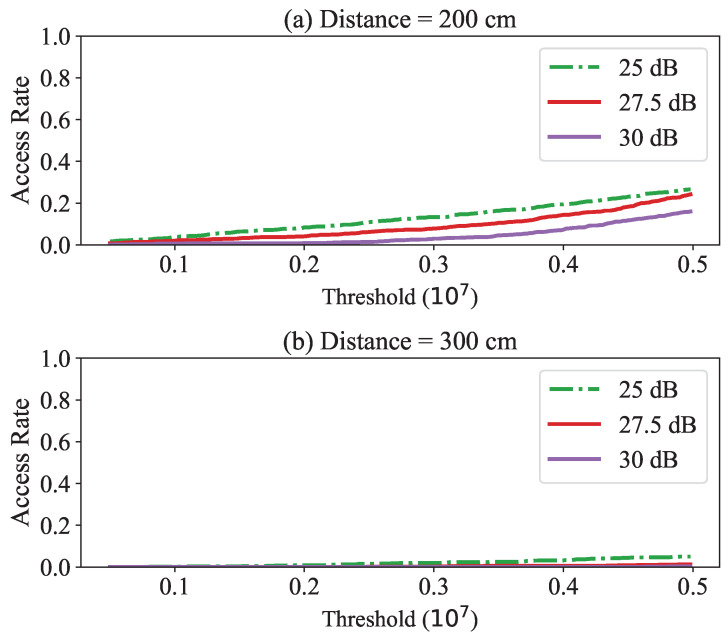
Access rate for different phase gradient threshold when the distance is near or far: (**a**) 200 cm and (**b**) 300 cm.

On the other hand, Figure 8 shows the access rate in the near or far state. In this case, since this was not a proximity situation, it was desirable for the access rate to be as low as possible. For example, at a distance of 200 cm, the acceptance rate was about 20% at the threshold, while the close (30 cm) access rate was 98%. Additionally, unlike the close situation, the false acceptance rate increased as the channel quality became worse.

Since the value of the phase gradient increased as the distance increased, the acceptance range could be reduced by lowering the threshold. In the case of 300 cm, even when the maximum value of the given threshold was used, the acceptance rate was only 0.3%, which shows that it would not pass *BackProx* in the case of a long distance. However, false acceptance may occur when the channel condition becomes worse due to the phase noise. A countermeasure to compensate for this is discussed in Section 7.

## 7. Discussion and Future Work

While *BackProx* is designed to not pass the prover when over 300 cm, it is possible that phase errors occur according to channel conditions, resulting in false acceptances. To address this issue, in the protocol described in Section 5.3, the error could be reduced by phase measurements totaling more than the mandatory two times. The average value of the phase difference is more tolerable in burst noise situations. Another approach to mitigate the error is to lower the threshold instead of the additional measurements. The threshold also affects the proximity range and the false acceptance rate. If the threshold is reduced, then it forces the user to be closer to the verifier and shrinks the proximity detection range.

The robustness of *BackProx* can be improved by cooperation with existing solutions. It is worth noting that the proposed method uses only the phase information provided by the existing system, so it can be used in conjunction with existing solutions such as distance-bounding protocols [15,16,17,44], encryption techniques, or using the radiometric signatures [26,45] to further enhance security.

One direction for future work is to investigate the possibility of an attacker being able to manipulate the response signals from LT and AT. Specifically, the attacker could inject different time delays into each signal, which makes it more difficult to measure the phase rotation at the verifier. This indicates a stronger adversarial model where the attacker can separate the response signals using specialized equipment with a high sampling rate, such as software-defined radios.

To this end, a more complex authentication process based on a multi-tag approach can be used. This requires hardware that is capable of analyzing the signals in the physical layer. Rather than measuring each tag individually, this approach imposes tag signal collisions between AT and LT signals and receives them simultaneously. Since the response signals arrives at the adversary simultaneously, this should inject the same time delay into the collided signal. This makes it impossible for the adversary to manipulate the response signal of LT or AT. This method has been used in previous studies such as [34,35], where it was used for simultaneous responses from multi-tagging to resist attacks such as tag counterfeiting or signal replay. We can adopt it to prevent attackers from separating the signals and inserting different time delays. On the other hand, with collision recovery techniques [46,47], the verifier can still obtain the phase information of each response signal, allowing application of the *BackProx* methodology.

## 8. Conclusions

In this paper, we proposed a novel secure proximity detection solution for backscattering networks called *BackProx*, which uses an additional backscattering tag to provide evidence of proximity. We examined three different types of relay attack models and provided security analysis on how *BackProx* effectively defends against these attacks. By using the phase difference between the legitimate tag and the additional tag, *BackProx* can successfully eliminate the time delay inserted by an adversary. We also provided a performance evaluation of *BackProx*. This showed that service providers can select their desired proximity boundaries by changing the threshold. The main advantage of *BackProx* is that it does not require any RF feature extraction or modification of the standard protocol, which makes it a practical solution for integrating into existing systems.

## Data Availability

Not applicable.

## Abbreviations

The following abbreviations are used in this manuscript:

AWGN	Additive white Gaussian noise
COTS	Commercial off-the-shelf
CW	Continuous wave
FHSS	Frequency hopping spread spectrum
GPS	Global positioning system
NFC	Near-field communication
PKES	Passive keyless entry and start
RF	Radio frequency
RFID	Radio frequency identification
RSSI	Received signal strength indication
SNR	Signal-to-noise ratio
ToF	Time of flight
